# The Effect of Chronic Exposure of Graphene Nanoplates on the Viability and Motility of A549 Cells

**DOI:** 10.3390/nano12122074

**Published:** 2022-06-16

**Authors:** Blanka Šestáková, Ladislava Schröterová, Aleš Bezrouk, Dana Čížková, Moustafa Elkalaf, Radim Havelek, Emil Rudolf, Věra Králová

**Affiliations:** 1 Department of Medical Biology and Genetics, Faculty of Medicine in Hradec Kralove, Charles University, Simkova 870, 500 03 Hradec Králové, Czech Republic; drdob9a1@lfhk.cuni.cz (B.Š.); rudolf@lfhk.cuni.cz (E.R.); kralovav@lfhk.cuni.cz (V.K.); 2 Department of Medical Biophysics, Faculty of Medicine in Hradec Kralove, Charles University, Simkova 870, 500 03 Hradec Králové, Czech Republic; bezrouka@lfhk.cuni.cz; 3 Department of Histology and Embryology, Faculty of Medicine in Hradec Kralove, Charles University, Simkova 870, 500 03 Hradec Králové, Czech Republic; cizkovad@lfhk.cuni.cz; 4 Department of Physiology, Faculty of Medicine in Hradec Kralove, Charles University, Simkova 870, 500 03 Hradec Králové, Czech Republic; elkalafm@lfhk.cuni.cz; 5 Department of Medical Biochemistry, Faculty of Medicine in Hradec Kralove, Charles University, Simkova 870, 500 03 Hradec Králové, Czech Republic; havelekr@lfhk.cuni.cz

**Keywords:** toxicity, nanomaterials, cell migration, pristine graphene, graphene accumulation, mitochondrial metabolism, in vitro, long-term cultivation

## Abstract

Graphene and its derivatives are popular nanomaterials used worldwide in many technical fields and biomedical applications. Due to such massive use, their anticipated accumulation in the environment is inevitable, with a largely unknown chronic influence on living organisms. Although repeatedly tested in chronic in vivo studies, long-term cell culture experiments that explain the biological response to these nanomaterials are still scarce. In this study, we sought to evaluate the biological responses of established model A549 tumor cells exposed to a non-toxic dose of pristine graphene for eight weeks. Our results demonstrate that the viability of the A549 cells exposed to the tested graphene did not change as well as the rate of their growth and proliferation despite nanoplatelet accumulation inside the cells. In addition, while the enzymatic activity of mitochondrial dehydrogenases moderately increased in exposed cells, their overall mitochondrial damage along with energy production changes was also not detected. Conversely, chronic accumulation of graphene nanoplates in exposed cells was detected, as evidenced by electron microscopy associated with impaired cellular motility.

## 1. Introduction

Graphene has been the most promising nanomaterial in the last decade in almost all fields of research. It has a unique honeycomb lattice structure enabling its exceptional Fphysical, chemical, and mechanical material properties such as superior electronic and thermal conductivity and optic sensitivity [1]. Graphene is a carbon nanomaterial allotrope formed of two-dimensional layers of molecules just one atom thick with a high surface-to-volume ratio. The unmodified pristine graphene with regular structure and high stability exhibits outstanding conductivity properties. Thus, it is a part of electrical conductors and nanoscaffolds used for sensors, batteries, conductors, displays, electronics, solar panels, etc. [2,3,4]. A disadvantage of this material is its poor solubility and tendency to aggregate in aqueous solutions [5]. There are several graphene derivatives obtained by the chemical functionalization of the graphene sheet’s surface, mainly with oxygen or nitrenes [6], that significantly modulate the reactivity properties of carbon-based materials and expand the diversity of their use [7]. Unlike pristine graphene, they can be easily dispersed in solutions but at the expense of their conductivity [8]. The most widespread graphene derivate is graphene oxide (GO). GO forms stable colloid suspensions in aqueous solutions and can be easily combined with other biomolecules and biomaterials. This favors its use in biomedical applications where GO conjugates with various drug molecules, antibodies, or ligands simultaneously may be employed for therapeutic or diagnostic purposes, such as in the case of photothermal therapy (PTT) and photodynamic therapy (PDT) [9]. Here, predominantly tumor cells were shown to spontaneously accumulate modified graphene sheets when injected intravenously into a mouse. After PTT, the tumor ablation was achieved with improved therapeutic efficacy and reduced toxic side effects [10].

Nanomaterials differ from their bulk parent materials in a number of characteristics, and their use in biomedical applications raises serious safety concerns. Graphene nanomaterials were shown to exert measurable toxicity both in vitro and in vivo [11]. At the same time, global graphene production is steadily rising, with graphene platelets being the basic building blocks for other derivatives in the graphene-family nanomaterials [12]. The risk of airborne graphene particles forming deposits in the lungs of workers has been the subject of several studies with inconclusive results [13,14,15,16]. Though in vivo studies are essential in evaluating the biological safety of nanomaterials, in vitro tests using mammalian cell lines are indispensable tools in cytotoxicity and genotoxicity studies and in elucidating the interactions of nanomaterials with target cells. Interestingly, existing in vitro studies mostly investigated the acute or short-term toxicity of nanomaterials, while there are only a few long-term toxicity data from chronic exposure experiments in vitro [12,17,18,19]. Therefore, this study aims to investigate the effects of chronic doses of graphene platelets in the A549 cell line in vitro.

## 2. Materials and Methods

### 2.1. Preparation of Nanomaterials

Graphene platelets 1 (GP1) were purchased from PlasmaChem GmbH (Berlin, Germany, product number PL-P-G750). The physicochemical characterization of GP1 is referred to in Table 1 [20].

Stock suspensions were prepared at a concentration of 250 µg/mL in 0.02% sodium cholate (Sigma-Aldrich, St. Louis, MO, USA) diluted in the ultrapure Milli-Q water using sonication and stored at room temperature in the dark [5,21]. For cell treatment, the stock solution was diluted in a culture medium to final concentrations of 5, 15, and 30 μg/mL. The osmolarity of each solution was checked by an osmometer, Osmomat 030 (Gonotec, Berlin, Germany).

### 2.2. Cell Culture and Treatment

The A549 cells were obtained from ATCC (Cat. No. CCL-185). They were maintained in αMEM without phenol red supplemented with 10% FSB, 1% antibiotics (penicillin, streptomycin), 10 mM Hepes, 1 mM sodium pyruvate, and 2 mM L-glutamine. The cells were kept at 37 °C and 5% CO_2_ atmosphere and subcultured twice a week.

**Scheme 1:**
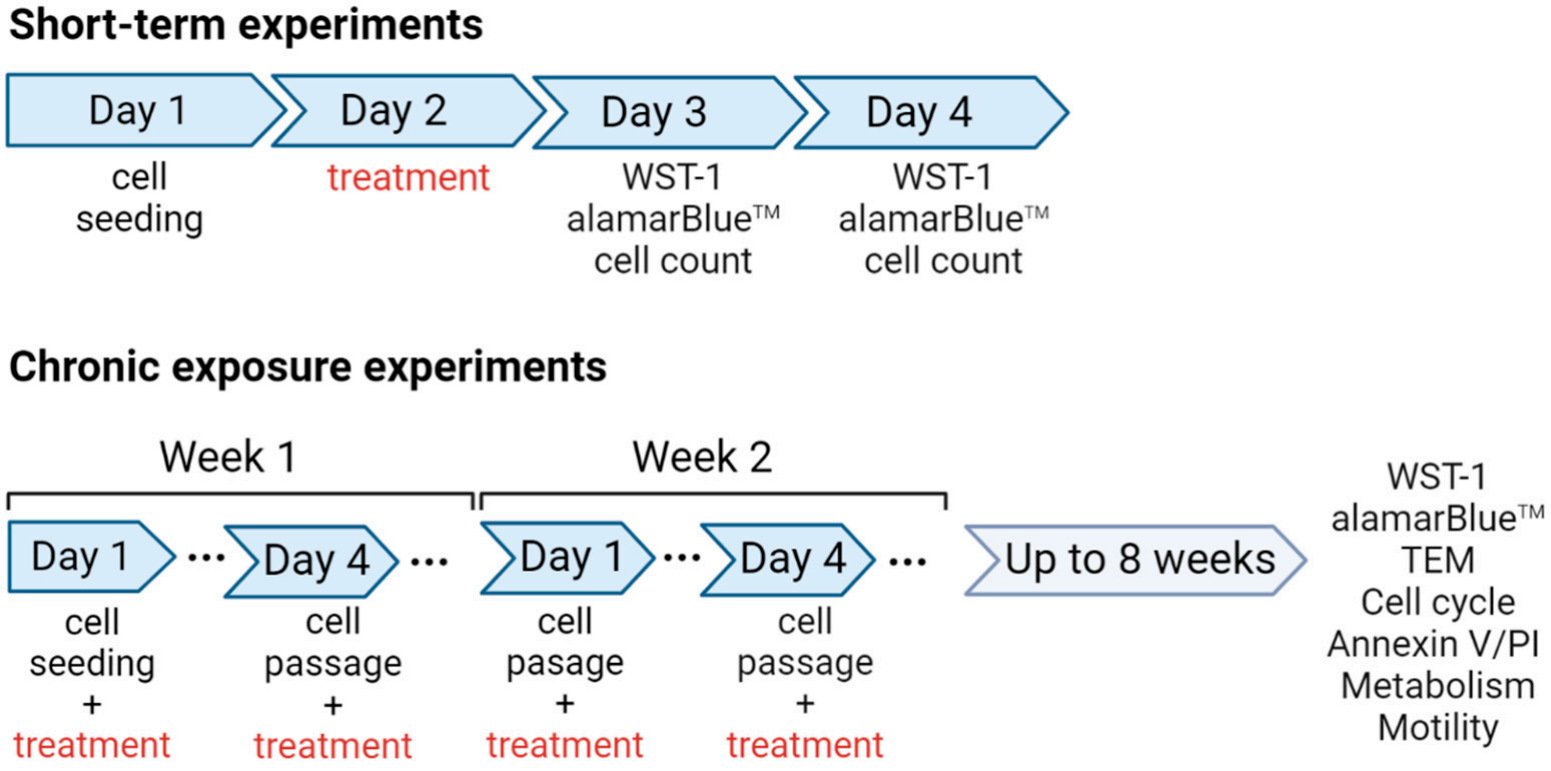
Treatment schedule of A549 cells.

For the acute cytotoxicity determination, 5000 cells per well were seeded in a 96-well plate in 100 μL of αMEM and incubated for 24 h. Then, the cells were exposed to graphene in concentrations of 5, 15, and 30 μg/mL and cultivated for 24 and 48 h (Scheme 1). Cytotoxicity assays were performed immediately after incubation.

For chronic exposure to GP1, the A549 cells were subcultured twice a week for a period of 8 weeks using the following protocol (Scheme 1): 1/10 of trypsinized cells were seeded in a new flask with fresh medium, allowed to adhere for 6 h, and then media were replaced with graphene-media mixture in concentrations of 5 μg/mL, 15 μg/mL or 30 μg/mL of graphene. Untreated control cells were subcultured using a normal culture medium in parallel. The control and graphene-treated cells were harvested at the end of the 8-week exposure. For subsequent metabolic assays using WST-1 and alamarBlue™ (Thermo Fisher Scientific, Waltham, MA, USA), 5000 cells per well were seeded in a 96-well plate in 100 μL of αMEM and incubated for 24 h. The rest of the freshly harvested cells were analyzed using cell cycle distribution analysis, Annexin V/propidium iodide cell death assay, and cell migration assays as described below.

### 2.3. Cytotoxicity Assays

The cytotoxicity of graphene nanomaterials was assessed by cell metabolic assays WST-1, alamarBlue™, and the total cell count using the automated imaging system, ImageXpress Micro XLS (Molecular Devices, San Jose, CA, USA). In the case of metabolic assays, the nanomaterials were first tested in acellular experiments to rule out possible interference of the materials with the assays.

#### 2.3.1. WST-1 Assay

After 24 h or 48 h treatment, 5 μL of the WST-1 solution (Roche, Basel, Switzerland) was added directly to the wells of the 96-well plate. Absorbance was recorded immediately (time 0 h) at 450 nm and 690 nm (reference wavelength) on the Tecan Infinite 200 PRO instrument (Tecan Trading) and then again after 1 h incubation. Final absorbance values were obtained by subtracting 1–0 h values.

##### 2.3.2. alamarBlue™ Assay

After 24 h or 48 h treatment, 10 μL of the alamarBlue™ (Invitrogen, Waltham, MA, USA) was added directly to the wells of the 96-well plate. Fluorescence was recorded immediately (time 0 h) at 560/590 nm fluorescence excitation/emission maxima on the Tecan Infinite 200 PRO instrument (Tecan Trading, Männedorf, Switzerland) and then again after 1 h incubation. Final fluorescence values were obtained by subtracting 1–0 h values.

### 2.4. Determination of Number Cell Nuclei by ImageXpress Micro XLS

After 24 h or 48 h treatment, the plate medium was replaced with 70% cold ethanol for 10 min. The cells were washed and stored in PBS at 4 °C until analysis. Immediately before the analysis, the cells were stained with DAPI (1 μg/mL final solution). Detection of fluorescent nuclei was performed on the ImageXpress Micro XLS system, and images were analyzed and quantified with the MetaXpress software (Molecular Devices, San Jose, CA, USA) using the multi-wavelength cell scoring application module.

### 2.5. Cell Cycle Distribution Analysis

Cells were harvested by trypsinization, collected, and washed with ice-cold Dulbecco’s phosphate-buffered saline (DPBS) (Sigma-Aldrich, St. Louis, MO, USA) and then fixed with 70% ethanol. The cells were centrifuged to remove ethanol and washed again with ice-cold DPBS. To detect low-molecular-weight fragments of DNA, the cells were incubated for 5 min at room temperature in a buffer (192 mL of 0.2 M Na_2_HPO_4_ + 8 mL of 0.1 M citric acid, pH 7.8) and then labeled with propidium iodide in Vindelov’s solution for 1 h at 37 °C. DNA content was determined by using the CytoFLEX LX Flow Cytometer (Beckman Coulter, Brea, CA, USA) with an excitation wavelength of 488 nm. The list-mode data were analyzed by Kaluza Analysis 2.1 software (Beckman Coulter, Brea, CA, USA).

### 2.6. Annexin V/Propidium Iodide Cell Death Assay

The cell death populations were determined by flow cytometry using an Alexa Fluor^®^488 Annexin V/Dead Cell Apoptosis kit (Life Technologies, Carlsbad, CA, USA) in accordance with the manufacturer’s instructions. The Alexa Fluor^®^488 Annexin V/Dead Cell Apoptosis kit employs the property of Alexa Fluor^®^488 conjugated to Annexin V to bind to phosphatidylserine in the presence of Ca2+, and the ability of propidium iodide (PI) to enter cells with damaged cell membranes and bind to DNA. Measurements were performed immediately after labeling using a CytoFLEX LX Flow Cytometer (Beckman Coulter, Brea, CA, USA). The list-mode data were analyzed using Kaluza Analysis 2.1 software (Beckman Coulter, Brea, CA, USA).

### 2.7. Chemotactic Migration Using the xCELLigence CIM-Plate-16

The lower wells of the CIM-Plate-16 were filled with 175 µL DMEM culture medium containing 10% fetal bovine serum. The upper wells were filled with 130 µL serum-free DMEM culture medium. The two parts of the plate were locked together and placed in the incubator according to the instructions of the manufacturer. After 1 h incubation, 100 µL of culture medium was removed from the upper wells and replaced with 100 µL of freshly harvested control and graphene-treated A549 cells in the serum-free medium. The seeding density was 4 × 104 cells per well. The plate was inserted in the xCELLigence RTCA-DP instrument (Agilent Technologies, Santa Clara, CA, USA), and the cells were allowed to migrate from the upper to the lower wells. The impedance was recorded every 10 min for a total of 24 h. The impedance values are displayed by the instrument as the cell index, which correlates positively with the number of cells that have migrated into the lower chamber.

### 2.8. Spontaneous Motility of the A549 Cells

The freshly harvested control and graphene-treated A549 cells were seeded into the 35 mm dish with a polymer coverslip bottom. The seeding density was 1 × 105 cells per dish in 2 mL of the DMEM culture medium supplemented with 10% fetal bovine serum. The cells were allowed to recover overnight, and the dish was then placed into the Biostation IM-Q (Nikon Instruments, Inc., Melville, NY, USA). The time-lapse video of the cells was recorded, with cells captured every 15 min for a total of 24 h. Cell motility was evaluated using the MTrackJ manual tracking plugin of the FIJI software [22]. A total of 100 control or graphene-treated cells were tracked. The mean velocity and the mean accumulated distance were calculated using the Chemotaxis and Migration Tool plugin for the FIJI Software (Chemotaxis and Migration Tool 2.0, Ibidi, Gräfelfing, Germany, free download from http://www.ibidi.de/applications/ap_chemo.html accessed on 12 July 2020).

### 2.9. Analysis of Metabolism

We used the Seahorse XFe-96 analyzer (Agilent Technologies, Santa Clara, CA, USA) to evaluate the effect of graphene on cellular metabolic function. Cells were treated for eight weeks with different concentrations of graphene, then harvested and seeded in the Seahorse XF cell culture microplates at a density of 15,000 cells per well one day prior to measurement. Before the measurement, we replaced the cell culture medium with the assay medium (bicarbonate-free XF DMEM Medium pH 7.4, Agilent Technologies, Santa Clara, CA, USA) supplemented with 4 mM L-glutamine, 1 mM pyruvate, and 1 g/L D-glucose. The cells were then incubated in a CO_2_-free incubator for one hour at 37 °C. After measuring the basal respiration, we performed a mitochondrial stress test by sequential additions of 1 μM oligomycin, 1.2 μM carbonyl cyanide-4-(trifluoromethoxy)phenylhydrazone (FCCP), and 1 μM rotenone-antimycin A mixture. The differences between OCR (oxygen consumption rate) values in response to respiratory modulators were used to calculate mitochondrial respiratory parameters such as ATP-related respiration and spare respiratory capacities. The changes in ECAR (extracellular acidification rate) were used to calculate glycolytic parameters (spare glycolytic capacity).

### 2.10. Analysis of Cell Morphology by Transmission Electron Microscopy (TEM)

The cells were processed for transmission electron microscopy as described previously [23]. Briefly, the cells were fixed in 3% glutaraldehyde (in 0.1 M cacodylate buffer, pH 7.2; Sigma) for 3 h at room temperature. Subsequently, the cells were washed in cacodylate buffer (0.1 M, pH 7.2) and post-fixed in 1% OsO4 (in 0.1 M cacodylate buffer, pH 7.2; Sigma) for 1 h at room temperature. After the rinsing procedure, the cells were dehydrated in graded alcohols (50%, 75%, 96%, and 100%), clarified in propylene oxide, and embedded in a mixture of Epon 812 and Durcupan (Sigma; polymerization for 3 days at 60 °C). Toluidine blue was used to stain the semithin sections. Ultrathin sections were cut on Ultrotome Nova (LKB, Vienna, Austria). These sections were then collected onto formvar carbon-coated copper grids, counterstained with uranyl acetate and lead citrate, and examined under a JEOL JEM-1400Plus transmission electron microscope (at 120 kV, JEOL, Tokyo, Japan). The images were taken with the integrated 8Mpix CCD camera and processed further using the software TEM Center (Ver. 1.7.3.1537, JEOL, Tokyo, Japan).

### 2.11. Statistical Analysis

The results represent the arithmetic means ± SD of three independent experiments. Significant differences between the tested groups in metabolic assays and the total cell counts were statistically evaluated by the One-way and Two-way ANOVA methods. The student’s *t*-test compared the differences between the tested groups in the viability tests and cell cycle analysis. Data from cell migration and spontaneous cell motility were evaluated by the Unpaired *t*-test. The GraphPad Prism 7 biostatistics (GraphPad Software, San Diego, CA, USA) software was used for all statistical analyses and graphs.

## 3. Results

### 3.1. Short-Term Cytotoxicity of Graphene

The viability of the A549 cells was evaluated after 24 h and 48 h exposure to GP1 nanomaterial using WST-1 and alamarBlue™ cytotoxicity assays. Non-specific nanomaterial interactions with assay chemistry were excluded in acellular assays prior to the testing (data in Supplementary Materials—Figure S1).

**Figure 1:**
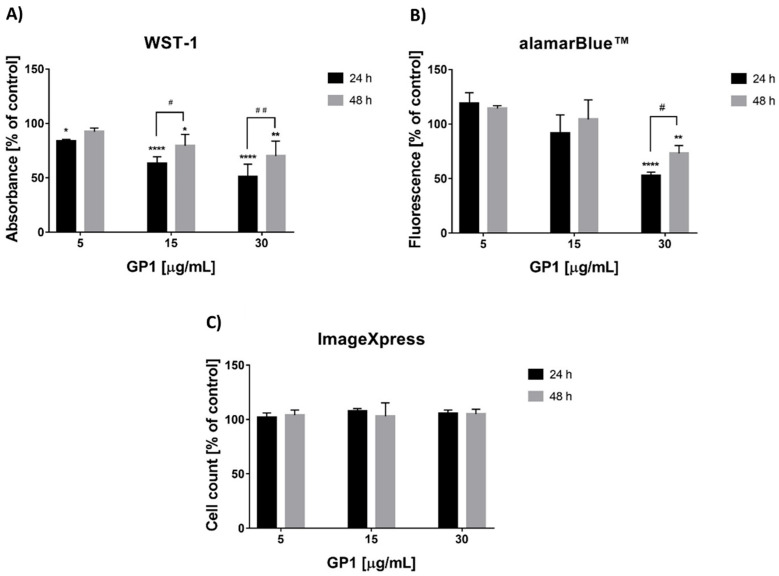
Effect of GP1 at concentrations of 5, 15 and 30 μg/mL on A549 cells during 24 h and 48 h as measured by cell metabolic assay—(**A**) WST-1, (**B**) cell metabolic assay—alamarBlue^TM^, (**C**) cell count performed on ImageXpress—IMEX. Data (optical density, cell count) represent the mean expressed as percentage of control (0 mM) ± SD of the three independent experiments. Statistical significance was evaluated with Two-way ANOVA Dunnett’s multiple comparisons test. Statistically significant decrease of values compared to controls * *p* < 0.05, ** *p* < 0.01, **** *p* < 0.0001. Statistically significant difference between two incubation times within one graphene concentration # *p* < 0.05, ## *p* < 0.01.

The GP1 nanomaterial caused a statistically significant dose-dependent decrease in metabolic activity after 24 h and 48 h in comparison to controls. On the other hand, a significant increase in metabolic activity of treated cells between 24 h and 48 h was apparent at both highest concentrations (Figure 1A).

Similar results were obtained using the alamarBlue™ method. The GP1-reduced metabolic activity at the highest concentration occurred only at both the 24 h and 48 h time intervals, with a significant difference between 24 h and 48 h (Figure 1B).

The total cell count was used to evaluate the effect of GP1 on cell proliferation. Cell nuclei were stained with DAPI, and their numbers in control and treated cells after 24 h and 48 h time intervals were determined with high-throughput image analysis using the ImageXpress Micro XLS system. The results did not show any significant difference in total cell count in GP1-treated cells in comparison to controls at both time intervals (Figure 1C).

### 3.2. Long-Term Effects of the GP1 Nanomaterial

#### 3.2.1. In Vitro Cytotoxicity

**Figure 2:**
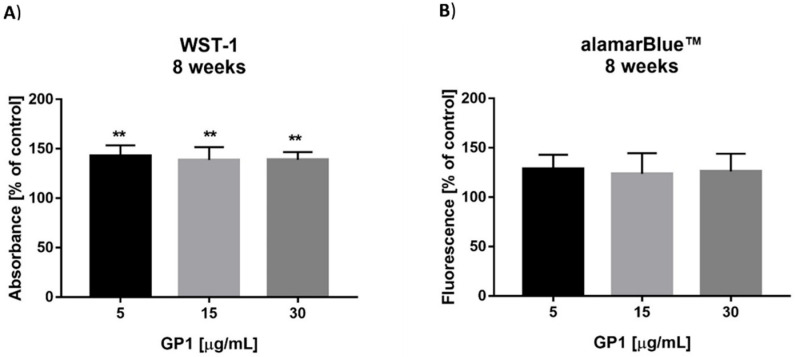
Long-term cytotoxic effect of graphene GP1 at concentrations of 5, 15, and 30 μg/mL on A549 cells during eight weeks as measured by cell metabolic assay—(**A**) WST-1, (**B**) cell metabolic assay—alamarBlue^TM^. Data (optical density, cell count) represent the mean expressed as percentage of control (0 mM) ± SD of the three different experiments. Statistical significance was evaluated with One-way ANOVA Dunnett’s multiple comparisons test. Statistically significant increase of values compared to controls ** *p* < 0.01.

The metabolic activity of the A549 cells exposed to GP1 twice a week during an eight-week treatment was measured using the WST-1 and alamarBlue™ assays. The GP1-treated cells showed elevated levels of metabolic activity, which was statistically significant in the case of the WST-1 assay, but not with the alamarBlue™ assay (Figure 2).

#### 3.2.2. Analysis of Metabolism

**Figure 3:**
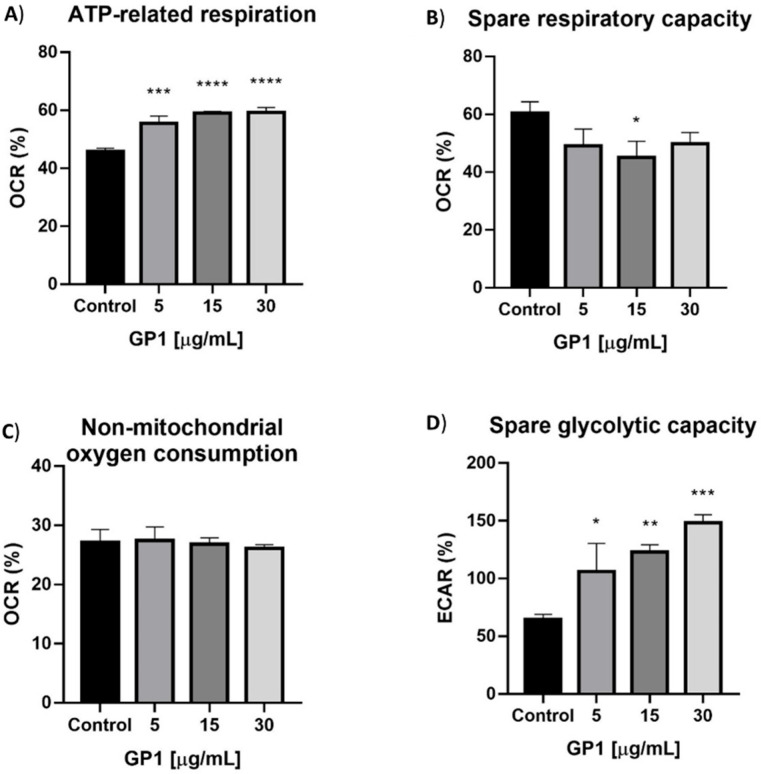
Analysis of metabolism after long-term treatment with GP1 in concentrations 5, 15, and 30 μg/mL using a Seahorse XFe96 analyzer (Agilent Technologies, Santa Clara, CA, USA). (**A**) ATP-related respiration, (**B**) spare respiratory capacity, (**C**) non-mitochondrial oxygen consumption, and (**D**) spare glycolytic capacity. Data represent the mean expressed as percentage of basal state ± SD of the three different experiments. Statistical significance was evaluated with One-way ANOVA Dunnett’s multiple comparisons test. Significant differences from control * *p* < 0.0332 ** *p* < 0.0021 *** *p* < 0.0002 **** *p* < 0.0001.

To investigate the effect of graphene on cellular metabolism, we used extracellular flux analysis to measure the cellular oxygen consumption rate (OCR) and the extracellular acidification rate (ECAR) as indicators of mitochondrial respiration and glycolysis. Graphene increased ATP-related respiration (basal OCR—OCR following inhibition of ATP synthase) in all treated groups (Figure 3A). The higher dependence on mitochondrial energy production did not affect mitochondrial maximal respiratory capacity (data not shown). Therefore, the spare respiratory capacity (maximal respiratory capacity—basal OCR) was marginally reduced in the treated groups (Figure 3B) as a plausible compensation for the stimulated ATP-driven respiration, while the cellular oxygen consumption for non-mitochondrial purposes was unchanged (Figure 3C). In addition, the long-term treatment with graphene stimulated cellular glycolysis and increased the spare glycolytic capacity (maximum ECAR following inhibition of mitochondrial ATP synthase—basal acidification rate) to reach more than a onefold increase with the highest graphene concentration (Figure 3D).

#### 3.2.3. Viability Analysis

**Figure 4:**
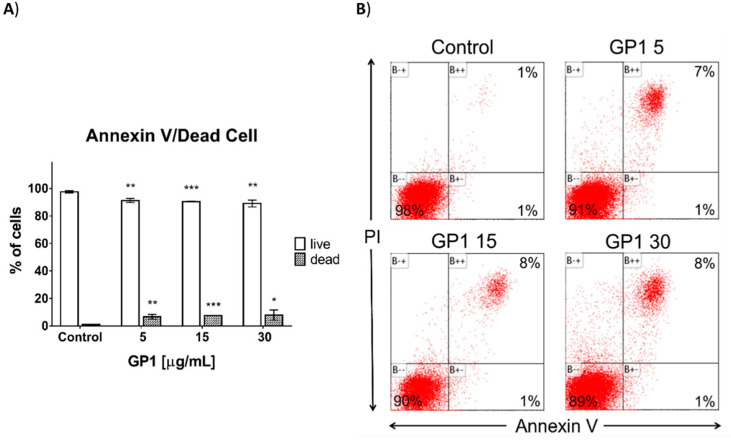
Viability analysis of A549 cells after eight-week treatment with GP1 in concentrations 5, 15, and 30 μg/mL using Annexin V/Dead Cell Apoptosis kit performed by flow cytometry. (**A**) Percentage of live and dead cells in control and GP1-treated cells (mean ± SD, *n* = 3). (**B**) Representative plots of one of the three experiments. Statistical significance was evaluated with Student’s *t*-test. Significant differences from control * *p* < 0.0332 ** *p* < 0.0021 *** *p* < 0.0002.

The live/dead cell ratio after an eight-week accumulation of GP1 in the A549 cells was determined with the Annexin V/Dead Cell Apoptosis kit using flow cytometry. Treated cells showed a significantly higher percentage of double-stained cells for Annexin V and propidium iodide in comparison with the untreated control: 0.97 ± 0.20% vs. 6.67 ± 0.98%, 7.55 ± 0.1%, 7.9 ± 2.15% resp. (Figure 4A). Almost all dead cells were double-stained with Annexin V and propidium iodide, which indicates necrotic death (Figure 4B), (B++ quadrants). Apoptotic death represented by cells stained with Annexin V only was insignificant and reached 1% in all samples (Figure 4B, B+− quadrants).

#### 3.2.4. Cell Cycle Distribution Analysis

**Figure 5:**
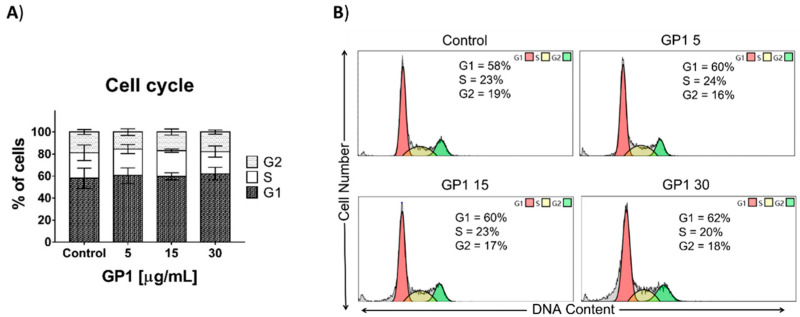
Analysis of cell cycle profile of A549 cells treated with GP1 for eight weeks as determined by flow cytometry. (**A**) Distribution of cell cycle phases (mean ± SD, *n* = 3). (**B**) Representative histograms of cell cycle analysis from one of three independent experiments. The percentage of individual cell cycle phases did not statistically differ between three independent experiments. Statistical significance was evaluated with Student’s *t*-test.

The distribution of cell cycle phases in A549 after the eight-week treatment with different concentrations of GP1 by flow cytometry was analyzed. The percentage of cells in individual phases of the cell cycle did not significantly differ between control and treated samples as well as among the three tested concentrations (Figure 5A). Representative results of one of the three independent experiments are shown in Figure 5B.

#### 3.2.5. Analysis of Cell Morphology by Transmission Electron Microscopy (TEM)

##### Acute Graphene Exposure

**Figure 6:**
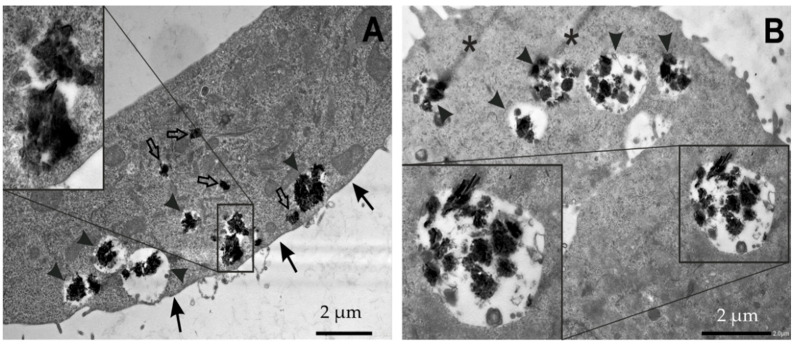
Representative TEM images of A549 cells confirm the internalization of graphene nanoplates. After the short-term exposition, (**A**) graphene nanoplates were located inside the cells, predominantly near the free surface of the cells (arrows). They form clusters that were fully or partly situated in the membrane-bound vesicles (arrowheads) or freely found in the cytoplasm (“empty” arrows). Following the 8-week exposition, (**B**) almost all graphene nanoplate clusters were observed inside the membrane-bound vesicles (arrowheads) in any parts of the cells. In comparison to the short-term exposition, vesicles containing graphene nanoplate clusters frequently reached bigger sizes. Smudges were arising from sectioning (asterisks). Scale bar A 2 µm (mag. 4000×), insert mag. 11,100×; B 2 µm (mag. 5000×), insert 7400×.

The majority of the A549 cells exposed to 30 µg/mL GP1 nanoplates for 48 h internalized them. Predominantly, the nanoplates were accumulated in membrane-bound vesicles, but smaller clusters were also observed in the cytoplasm without a delimitating membrane. The vesicles with accumulated nanoplates were often situated in the vicinity of the free surface of the cultivated cells, whereas smaller nanoparticle clusters were recorded throughout the cytoplasm (Figure 6A). Nanoplates were not found in the cell nuclei. The cell ultrastructure did not reveal any pathological changes.

##### Long-Term Graphene Exposure

The GP1 30 graphene nanoplates were observed nearly in all of the A549 cells inside the membrane-bound vesicles only after the long-term exposure. The vesicles increased in their size compared to the short time exposure and occurred in any cell cytoplasm area. They were not found in the cell nuclei.

#### 3.2.6. Effect of GP1 on A549 Cell Migration

**Figure 7:**
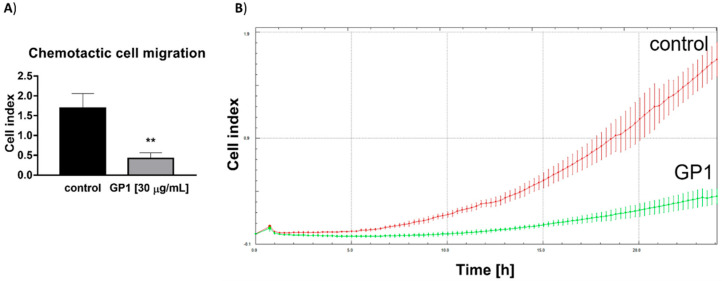
(**A**) Cell index of control and graphene-treated cells after 24 h migration (**A**). Statistical significance was evaluated with an Unpaired *t*-test. ** significant difference from control cells, *p* < 0.05. (**B**) Representative migration curves of control and GP1-treated cells from three independent experiments.

The A549 cells treated with 30 µg/mL GP1 for eight weeks showed a significantly decreased migration curve in comparison with untreated controls. While the cell index of the controls at 24 h reached 1.69 ± 0.37, the GP1-treated cells showed only 0.42 ± 0.14 (Figure 7A,B demonstrate a representative result from three independent measurements).

#### 3.2.7. Effect of GP1 on Spontaneous A549 Cell Motility

**Figure 8:**
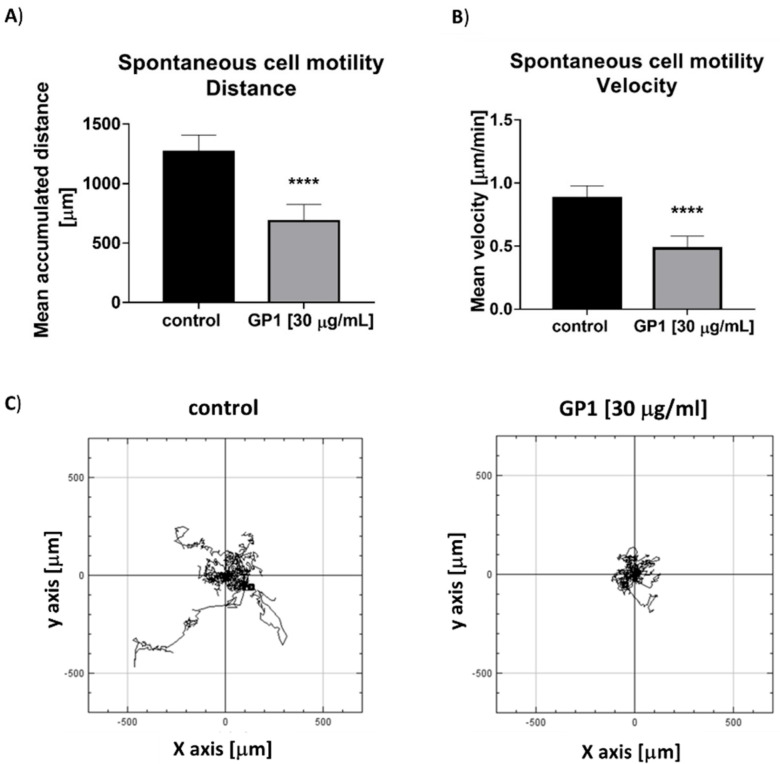
Mean accumulated distance (**A**) and mean velocity (**B**) of GP1-treated cells and controls over the course of 24 h. Statistical significance was evaluated with an Unpaired *t*-test. **** significantly different from controls, *p* < 0.0001. Graphical representation of spontaneous cell motility using the Chemotaxis Tool, representative result from one of the measurements (**C**).

Time-lapse observations revealed that the A549 cells treated with 30 µg/mL GP1 were significantly slowed down in their spontaneous motility on the surface of the culture dish. Their average velocity calculated over 24 h was 0.48 ± 0.1 µm/min, and the average accumulated distance per cell was 685.1 ± 138.9 µm, compared to 0.88 ± 0.1 ± µm/min and 1266.7 ± 141.3 µm in the control cells (Figure 8).

## 4. Discussion

The alveolar region of the lung is of particular interest to those studying the effects of airborne materials with diameters lower than 2 μm. In vitro lung cell cultures offer a cost-effective and robust platform for performing in vitro toxicology studies [24]. An A549 non-small cell lung carcinoma cell line is a well-characterized standard system that has been widely used as a model for type II pulmonary epithelial cells as well as a model of lung adenocarcinoma [25,26] and has been investigated for its long-term stability and inter-laboratory variability [24]. As such, A549 cells were already employed in short-term toxicity studies of nanomaterials, including graphene derivatives and their combinations with other nanomaterials [27,28,29,30]. In our study, the sensitivity of the A549 cells to graphene nanoplates during the short-term exposure was dependent on the type of assay. The WST-1 assay was more sensitive than the alamarBlue™ assay, while direct counting of nuclei using high-throughput image cytometry did not reveal any toxicity of the GP1 nanoplates. The WST-1 assay tetrazolium salt is cleaved to a soluble formazan by a complex cellular mechanism that occurs primarily at the cell surface and was reported to yield false-positive results because of the optical interference between nanomaterials and tetrazolium salts and other suggested modes of interactions [31,32,33]. Resazurin in alamarBlue™ is reduced by enzymes located in cytoplasm and mitochondria, and its reduction may signify an impairment of cellular metabolism without necessarily implicating mitochondrial dysfunction [34]. A cross-reactivity check with tested compounds has been recommended for alamarBlue™ [31]. In this study, we did not observe any interference in the acellular experiments using the WST-1 (Figure S1A). However, in the case of the alamarBlue™, the highest concentration of GP1 caused a decrease in fluorescence in acellular experiments in comparison to the controls (Figure S1B), which might be caused by fluorescence quenching.

Next, we investigated the effects of GP1 in a chronic exposure test. Published data on the chronic exposure of cells to nanomaterials in vitro have been rather limited so far. In the case of gold nanoparticles, it was reported that the long-term, 20-week chronic exposure of cells did not affect cell viability, which remained over 92%. Interestingly, this study compared cellular response after a single initial dose followed by a 20-week cultivation in the absence of gold nanoparticles to a chronic 20-week exposure. It was found that the cells could adapt to chronic doses while being significantly more influenced by acute insult even after a 20-week period [17]. The A549 cells, after an eight-week cultivation in the presence of chronic GP1 doses, showed an increase in metabolic activity in comparison to the untreated controls, which was statistically significant using the WST-1 assay but not with the alamarBlue™ assay, which again indicates the higher sensitivity of the WST-1 assay in comparison to the alamarBlue™ assay in this study. The cells showed similarly elevated levels of WST-1 reduction regardless of the GP1 concentration used. This increase in metabolic activity may represent an adaptive mechanism to continuous stress [35]. The viability test based on the Annexin V/propidium iodide staining revealed a significant but moderate increase in the percentage of dead cells in comparison to untreated controls, regardless of tested concentration. These results suggest the involvement of the necrotic cell death since the dead cells were double positive for Annexin V and propidium iodide. At the same time, the cell population was actively proliferating, as the analysis of cell cycle distribution did not reveal any significant changes in comparison with the untreated controls.

Cellular uptake of graphene nanomaterials depends on their physicochemical properties as well as on the cell type investigated [36] or the cell differentiation status [37]. Graphene and its derivatives are supposed to enter cells via endocytosis or by mechanical penetration due to their sharp edges [38]. In the study of Chang et al., graphene oxide was not found internalized within the A549 cells [27], and similarly, glioblastoma cells did not seem to take up graphene platelets [39]. Conversely, Jin et al. found that graphene oxide entered the A549 cells by cellular uptake and was located in the cytoplasm and the nucleus, not causing any decrease in cell viability [28]. In this study, transmission electron microscopy clearly demonstrated the presence of GP1 within the A549 cells after an eight-week exposure. Our data also suggest that the cells tended to enclose the GP1 clusters that were originally present freely in the cytoplasm into membrane-bound vesicles and that these vesicles tended to increase in their size during the eight-week exposure. This may be the result of activated autophagy as a cytoprotective mechanism. Nanoparticles were shown to affect autophagy significantly, increasing the autophagosome formation and blocking the autophagic flux [18,40]. Regarding the graphene-based nanoparticles, graphene oxide was the most investigated form with respect to autophagy, inducing the autophagic process in various cell lines [41,42,43]. Graphene platelets were reported to induce autophagy in a dose-dependent manner in BEAS-2B cells [44]. Further experiments will be needed to investigate the possible involvement and role of autophagy in our long-term experimental settings in A549 cells.

In contrast to the low toxicity demonstrated by the above-mentioned assays, we found a significant impact of chronic GP1 exposure on cell motility. In a transwell assay using the impedance-based measurements, the GP1 in 30 µg/mL concentration significantly reduced the number of cells that migrated into the lower wells. Cell migration in transwell assays is driven by chemotaxis, so the effects of GP1 on the A549 cells could involve both impairments of structures necessary for motility or interference with chemotactic signaling. We, therefore, also investigated spontaneous 2D motility of the A549 cells in culture using time-lapse microscopy followed by cell tracking. The GP1 in 30 µg/mL concentration significantly decreased both the mean cell velocity and mean accumulated distance of the A549 cells during the 24 h observations. Cellular motility is essential for vital mechanisms such as development, wound healing, and immune system functions, but also for invasiveness and metastatic behavior of malignant cells. Interestingly, reduced graphene oxide (rGO) was reported to induce epithelial-mesenchymal transition (EMT) in A549 cells while significantly reducing their migration at 20 µg/mL. However, the increase in cell migration potential is considered to be one of the EMT characteristics [29]. On the contrary, human RPE cell migration was enhanced by graphene oxide during the EMT process [45]. Precise causes of the underlying impairment of motility by graphene and its derivatives remain to be elucidated. In an acellular study, graphene flakes were shown to increase the length and the elongation rate of actin filaments without hampering bulk actin polymerization [46]. Other mechanisms have been proposed, such as the involvement of ROS [47,48], signaling via chemokines and cytokines [49], or direct adhesion of actin monomers to graphene by weak interactions [50]. In a study involving normal and cancer cells, graphene and graphene oxide interfered with the electron transport chain in mitochondria, which resulted in a decrease in energy production and any impaired assembly of the actin cytoskeleton and lamellipodia formation [51]. In contrast, our analysis of long-term GP1 effects on cellular metabolism in A549 cells does not indicate impairment in energy production. Cell migration is also an essential part of cell division, during which the two daughter cells move apart. The timelapse sequences of GP1-treated A549 cells, even after eight weeks, portrayed dividing cells, which corresponds to the results of cell cycle analysis. We attempted to track cells undergoing cytokinesis and observed a very similar decrease in daughter cell velocity (Figure S2A) and accumulated distance (Figure S2B) as with the interphase cells. Such phenomenon evidently does not interfere with the cell cycle progression of the GP1-loaded A549 cells in vitro but might impair the regeneration and wound-healing capacity of affected tissues in vivo since the balance between proliferation and migration, and their mutual coordination is essential for the proper execution of these regenerative processes [52].

In conclusion, we evaluated the effects of chronic eight-week exposure to graphene platelets in the A549 cells. Our results show for the first time that the cells in the presence of graphene platelets were able to survive and proliferate for prolonged time periods, with graphene platelets accumulated in membrane-bound vesicles, slightly elevated metabolic activity, and a moderate increase in cell death. Graphene platelets did not affect cellular proliferation. Their major observed effect was impairment in cellular motility, which was not likely attributable to a deficiency in cellular energy production. These preliminary results raise important questions about fate and possible long-term effects of graphene-loaded cells within an organism. Follow-up research is also needed involving normal, non-cancerous cells to elucidate molecular mechanisms involved in cellular stress response against graphene platelets during chronic exposures.

## Figures and Tables

**Table 1:** Physical properties of GP1.

Suspension Diluted in	Particle Size (nm) (Z-Average)	PdI	Average *ζ*-Potential (mV)
Sterile water	179 ± 103	0.188	−42.5 ± 6.9
Full medium	208 ± 103	0.203	−8.52 ± 15.3

## Data Availability

Data is contained within the article.

## Supplementary Materials

The following supporting information can be downloaded at: https://www.mdpi.com/article/10.3390/nano12122074/s1, Figure S1A: Acellular test WST-1; Figure S1B: Acellular test alamarBlue^TM^ Figure S2A: Cytokinesis of the A549 cells—velocity, Figure S2B Cytokinesis of the A549 cells—accumulated distance.
