# Supplementary material for: The Effect of Chronic Exposure of Graphene Nanoplates on the Viability and Motility of A549 Cells
Source: Nanomaterials (Basel). 2022 Jun 16;12(12):2074. doi: 10.3390/nano12122074 (PMC9227066; doi:10.3390/nano12122074)
Supplement: Supplementary file 1 [file nanomaterials-12-02074-s001.zip › nanomaterials-1769113-supplementary.pdf]

nanomaterials

Supplementary materials

The Effect of Chronic Exposure of Graphene Nanoplates on the Viability and Motility of A549 Cells

Blanka Šestáková [1], Ladislava Schröterová [1,*], Aleš Bezrouk [2], Dana Čížková [3], Moustafa Elkalaf [4], Radim Havelek [5], Emil Rudolf [1] and Věra Králová [1]

1 Department of Medical Biology and Genetics, Faculty of Medicine in Hradec Kralove, Charles University, Simkova 870, 500 03 Hradec Králové, Czech Republic; drdob9a1@lfhk.cuni.cz (B.Š.); rudolf@lfhk.cuni.cz (E.R.); kralovav@lfhk.cuni.cz (V.K.)
2 Department of Medical Biophysics, Faculty of Medicine in Hradec Kralove, Charles University, Simkova 870, 500 03 Hradec Králové, Czech Republic; bezrouka@lfhk.cuni.cz
3 Department of Histology and Embryology, Faculty of Medicine in Hradec Kralove, Charles University, Simkova 870, 500 03 Hradec Králové, Czech Republic; cizkovad@lfhk.cuni.cz
4 Department of Physiology, Faculty of Medicine in Hradec Kralove, Charles University, Simkova 870, 500 03 Hradec Králové, Czech Republic; elkalafm@lfhk.cuni.cz
5 Department of Medical Biochemistry, Faculty of Medicine in Hradec Kralove, Charles University, Simkova 870, 500 03 Hradec Králové, Czech Republic; havelekr@lfhk.cuni.cz
* Correspondence: schroteroval@lfhk.cuni.cz; Tel.: +420-495-816-284

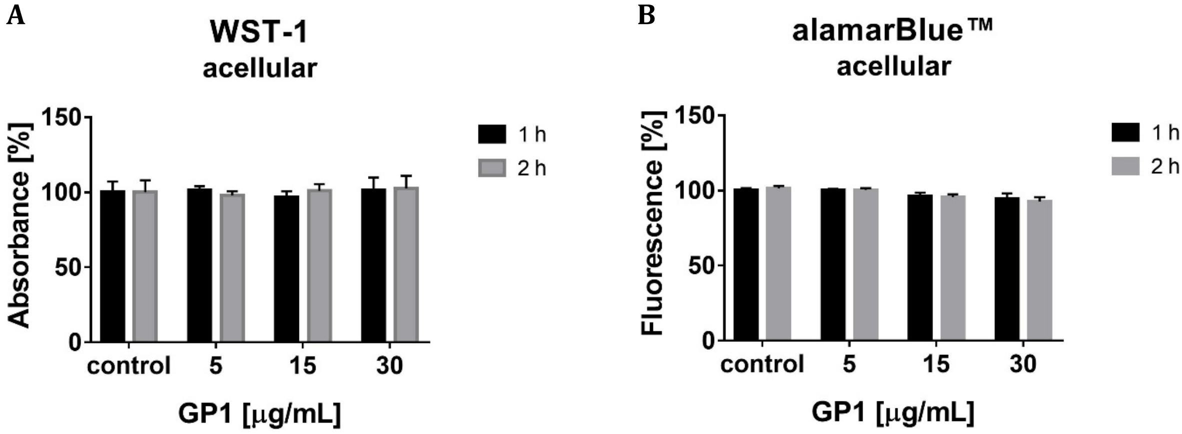

Figure S1. (A) Acellular test WST-1; (B) Acellular test alamarBlue™.

Acellular tests were performed to check possible interference of tested graphene nanoplates with the WST-1 and alamarBlue™ reactions. The WST-1 or the alamarBlue™ substrates were added to culture medium containing 0, 5, 15 and 30 µg/mL GP1. Depending on the method, absorbance (WST-1) or fluorescence (alamarBlue™) was measured at times 0 (background), 1 and 2h. Graphs show absorbance or fluorescence values at 1h and 2h, after subtracting the background. Absorbance values did not differ significantly from controls (A). Fluorescence values decreased by a maximum of 8% at the highest tested concentration of graphene compared to controls (B).

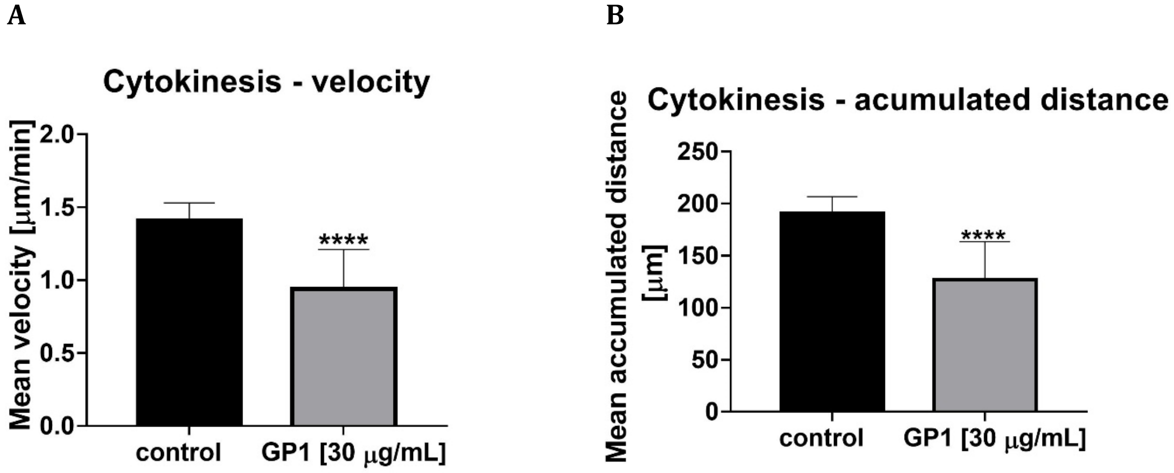

Figure S2. (A): Cytokinesis of the A549 cells – velocity, (B) Cytokinesis of the A549 cells – accumulated distance.

Freshly harvested control and graphene-treated A549 cells were seeded into the 35-mm dish with a polymer coverslip bottom. The seeding density was 1 x 10^5 cells per dish in 2 mL of the DMEM culture medium supplemented with 10% fetal bovine serum. The cells were allowed to recover overnight and the dish was then placed into the Biostation IM-Q (Nikon Instruments, Inc., Melville, NY 11747-3064, U.S.A.). The time-lapse video of the cells was recorded, with cells captured every 15 min for a total of 24 h. Motility of daughter cells during cytokinesis was evaluated using the MTrackJ manual tracking plugin of the FIJI software. Cells were tracked from the start of anaphase for 10 time points. A total of 95 control and 63 graphene-treated cells were tracked. The mean velocity (A) and mean accumulated distance (B) were calculated using the Chemotaxis and Migration Tool plugin for the FIJI Software (Chemotaxis and Migration Tool 2.0, Ibidi, free download from http://www.ibidi.de/applications/ ap_chemo.html). The graphene-treated A549 cells were significantly slowed down during cytokinesis in comparison to the untreated cells. Their mean velocity calculated over 10 time points was 126.9 ± 36.6 μm/min and average accumulated distance per cell was 0.94 ± 0.27 μm, compared to 190.9 ± 15.8 μm/min and 1.41 ± 0.12 μm in the control cells.
